# Medical Courtship

**Published:** 1889-07-27

**Authors:** 


### Medical Courtship.

A man of modern science wooed
A maiden of accepting mood,
Who, dreading lest contagion might
Do mischief to her chosen wight,
With sol. bichloride washed her hair
And sponged her limbs and body fair.

She rinsed her mouth with Listerine,
And held, her snow-white teeth between,
A pad of antiseptic gauze,
Covering her nose, as well as jaws,
Which formed a sort of respirator
Between them and her osculator.

But this reminds: I should have told
That these were things he'd taught of old,
With others which I may not tell, in
Regard to spots that germs might dwell in.
She was a wise professor's daughter
And practised all which had been taught her.

So this good medicine man, with pride
Clasping his antiseptic bride,
In disinfected murmur low
Asked, "Why she loved her doctor so?"
And, softly nestling down, she sighed,
"You're such a dear old germicide."

From the *Medical and Surgical Journal.*

